# Accidental Dural Tears in Minimally Invasive Spinal Surgery for Degenerative Lumbar Spine Disease

**DOI:** 10.3389/fsurg.2021.708243

**Published:** 2021-07-20

**Authors:** Stefan Aspalter, Wolfgang Senker, Christian Radl, Martin Aichholzer, Kathrin Aufschnaiter-Hießböck, Clemens Leitner, Nico Stroh, Wolfgang Trutschnig, Andreas Gruber, Harald Stefanits

**Affiliations:** ^1^Department of Neurosurgery, Kepler University Hospital, Johannes Kepler University, Linz, Austria; ^2^Department of Mathematics, University of Salzburg, Salzburg, Austria

**Keywords:** spine, spinal fusion, spine surgery, minimally invasive surgical procedure, cerebrospinal fluid leak, dural tear

## Abstract

**Background:** One of the most frequent complications of spinal surgery is accidental dural tears (ADTs). Minimal access surgical techniques (MAST) have been described as a promising approach to minimizing such complications. ADTs have been studied extensively in connection with open spinal surgery, but there is less literature on minimally invasive spinal surgery (MISS).

**Materials and Methods:** We reviewed 187 patients who had undergone degenerative lumbar spinal surgery using minimally invasive spinal fusions techniques. We analyzed the influence of age, Body Mass Index (BMI), smoking, diabetes, and previous surgery on the rate of ADTs in MISS.

**Results:** Twenty-two patients (11.764%) suffered from an ADT. We recommended bed rest for two and a half to 5 days, depending on the type of repair required and the amount of cerebrospinal fluid (CSF) leakage. We could not find any statistically significant correlation between ADTs and age (*p* = 0.34,), BMI (*p* = 0.92), smoking (*p* = 0.46), and diabetes (*p* = 0.71). ADTs were significantly more frequent in cases of previous surgery (*p* < 0.001). None of the patients developed a transcutaneous CSF leak or post-operative infection.

**Conclusions:** The frequency of ADTs in MISS appears comparable to that encountered when using open surgical techniques. Additionally, MAST produces less dead space along the corridor to the spine. Such reduced dead space may not be enough for pseudomeningocele to occur, cerebrospinal fluid to accumulate, and fistula to form. MAST, therefore, provides a certain amount of protection.

## Introduction

Minimally invasive access surgery techniques (MAST) are associated with reduced blood loss, faster recovery, and reduced perioperative morbidity while yielding similar results to open procedures (1–4). Minimally invasive spinal surgery (MISS) is believed to provide a smaller corridor to the spine and result in minor tissue injury. For minimally invasive lumbar spine surgery, endoscopic techniques (5) and techniques using tubular retractor systems were introduced. With the introduction of tubular retractor systems, it was possible to perform less muscle dissection and better cosmetic incisions, which can lead to decreased pain and faster recovery after surgery. Limitations of the usage of tubular retractors are increased costs and a steeper learning curve (6). Accidental dural tears (ADTs) are an unpleasant but not infrequent intraoperative complication. Some of the issues generally associated with accidental dural tears are secondary scar tissue, hypertrophy, and secondary ossification of the yellow ligament, as well as synovial cysts (7). However, intraoperative complications, especially ADTs, have scarcely been reported in connection with minimally invasive spinal procedures. The frequency of ADTs in MISS is reported to range from 3.2 to 16.7% (7–11). This prospective study aimed to examine whether patients who undergo minimally invasive surgery (MIS) of the spine are at a higher risk of experiencing an ADT. As part of this study, we investigated whether age, obesity, smoking, diabetes, and previous surgery impact the likelihood of ADT occurrence. To our knowledge, this is one of the largest single-center studies.

## Materials and Methods

After obtaining approval for the study from the ethics committee of the Federal State of Lower Austria, we recruited 187 patients for this prospective investigation. One hundred fifteen of these were female and 72 male. Informed consent was obtained from all patients. We registered the study on http://www.clinicaltrials.gov (ID: NCT01259960).

We performed Lumbar MIS fusion using interbody fusion procedures and posterolateral fusion alone. All patients had been treated with one, two, three, or four-level minimally invasive fusion for degenerative diseases of the lumbar spine. In spinal stenosis cases, we performed additional decompression of the spinal canal. We collected data on the patients' age, gender, height, weight, smoking status, and presence of diabetes. Type of procedure, the occurrence of accidental dural leaks, and other intra- and post-operative complications were recorded.

Considering accidental dural tears, we analyzed if age, BMI, smoking, diabetes, and previous surgery had a significant influence on the rate of ADTs. Regarding age, patients were grouped into four cohorts: 33–55, 56–65, 66–75, and 76–85 years. A standard four-sample test for equality of proportions was performed considering the ADT rate within the four groups. Regarding BMI, cohorts were made according to the WHO classification. BMI is calculated by dividing the subject's mass by the square of the person's height (BMI = kg/m^2^). Individuals with a BMI <25 are considered to be of normal weight, those with a BMI >25 and <30 overweight, and those with a BMI >30 obese. Testing for significant differences between the BMI groups was performed using a standard three-sample test for equal proportions. Testing of significant differences in the smoker/non-smoker, diabetes/no-diabetes, and previous surgery/no previous surgery groups was performed using standard two-sample tests for equality of proportions.

### Surgical Technique

**Figure 1:**
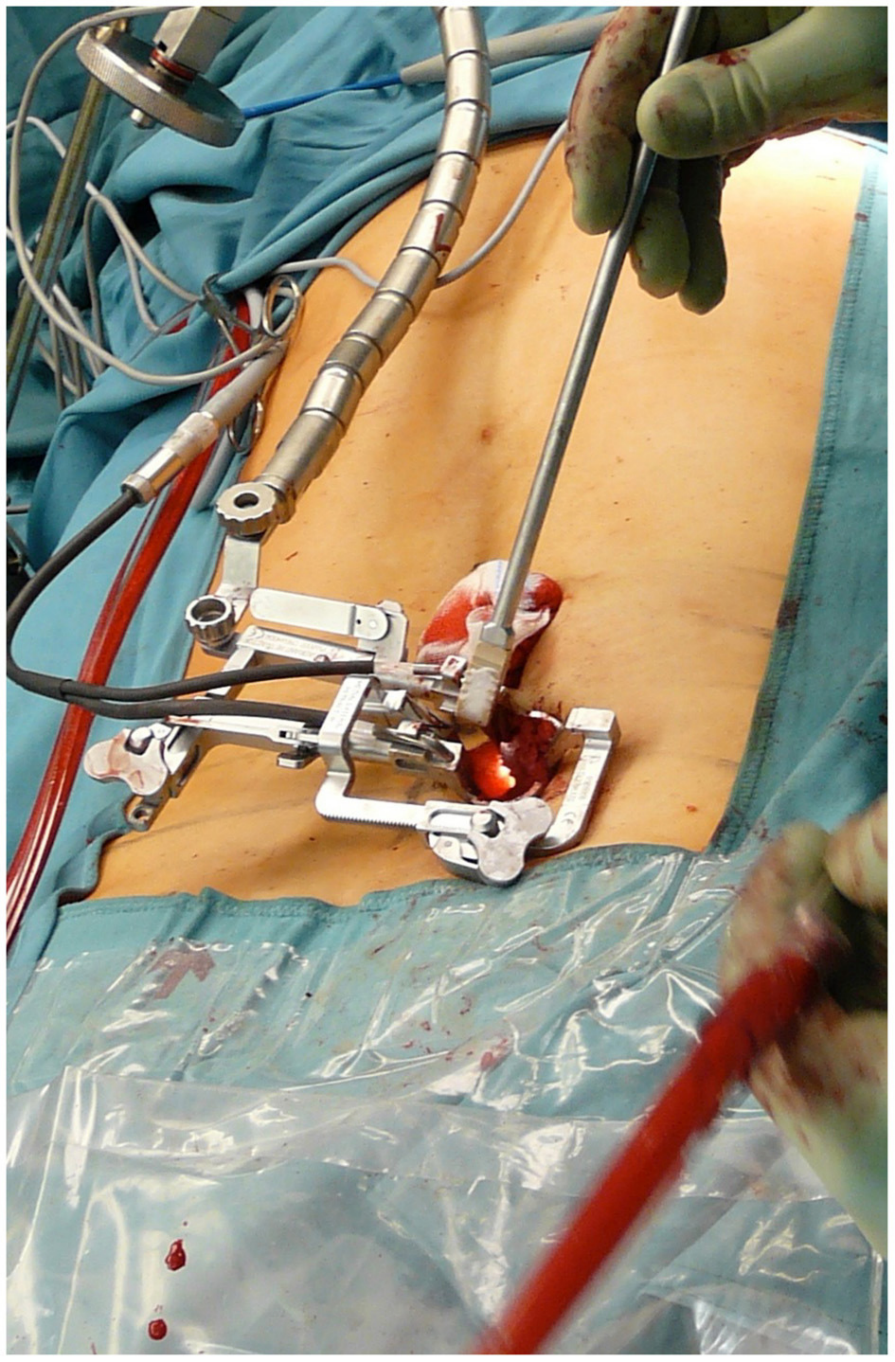
Implantation of a TLIF cage in a minimally invasive procedure using a tubular retractor.

In all cases, we performed a minimally invasive approach using the Quadrant Tubular Retractor system (Medtronic Inc., Memphis, TN). Two surgeons performed the operations. In 360° fusion cases, a TLIF procedure was performed (12). In spinal stenosis cases, laminotomy, and revision of the disc space were performed. Under fluoroscopy, we identified the relevant facet joint, and a paramedian incision was made. By sequential dilating the subcutaneous and muscle tissue, a corridor was created to the facet joint, similar to the technique described by Foley and Smith (13). The tubular retractor was then inserted, and after identification of the anatomical landmarks, we resected the facet joint and the ligamentum flavum. In spinal stenosis cases, the retractor was directed to the contralateral side of the spinal canal to perform laminotomy to provide sufficient decompression of the spinal canal. The intervertebral disc was then identified and resected, and the endplates were prepared for implantation of the cages. Figure 1 shows the implantation of a TLIF cage. We used the percutaneous fusion system Sextant II or Longitude (both Medtronic Inc., Memphis, TN). In instances where durotomy occurred, we tried to close the dura with 6–0 or 7–0 Prolene (Ethicon, Somerville, NJ, USA). If direct closure could not be achieved, we tried to fix the defect with a hemostatic patch (TABOTAMP^®^, Johnson & Johnson Medical Ethicon Biosur, Somerville, NJ, USA) and DuraSeal® (Integra Lifesciences Corporation, Plainsboro, NJ, USA). In all cases, an absorbable hemostatic gelatin sponge (SPONGOSTAN, manufactured by Ferrosan A/S, Soeborg, Denmark; distributed by Johnson & Johnson, New Brunswick, NJ, USA) and fibrin glue were applied to the corresponding vertebral arch to prevent the formation of cerebrospinal fluid fistulas. Post-operatively, we recommended bed rest for two and a half to 5 days, depending on the size of the defect and whether primary closure had been performed.

## Results

The full dataset consisted of 187 patients who had undergone surgery. Patient baseline characteristics are provided in Table 1. One hundred fifteen patients were female and 72 male. The mean age across the total sample was 64.27 years, ranging from 33 to 85 years. Forty-five patients each were in the age groups 33–55, 56–65, and 66–75 years. Fifty-two patients were allocated to the group of 76–85 years. Thirty patients from our sample were of normal weight, 79 overweight and 78 obese. The age distribution per BMI group did not differ significantly (*p* = 0.7). Twelve patients (6.15%) of the entire cohort had had prior surgery. We performed additional decompression of the spinal canal in 146 patients. Ninety-seven (51.87%) patients underwent mono-segmental minimal invasive fusion, and 90 patients multi-level fusion (two segments in 61 cases, three segments in 22 cases, and seven segments in 4 cases). We saw liquor leakages in 22 patients (11.764%). All ADTs occurred during preparation and decompression of the spinal canal using surgical instruments like Kerrison rongeurs. Table 2 shows the rate of accidental dural tears (ADTs) in total and by patient subgroups. Four of these patients were aged between 33–55, six between 56–65 years, eight between 66–75 years, and four between 76–85 years. No significant difference in the ADT rate between the groups was found (*p* = 0.34). Three patients of the ADT group were in the normal weight BMI group, nine patients in the overweight group, and 10 patients in the obese group, and the standard three-sample test for equality of proportions yielded a *p*-value of 0.92, and therefore no significant difference between the weight groups. In the smoking group, four liquor leakages occurred, while the remaining 18 ADTs occurred in non-smoking patients. With a *p*-value of 0.46, the difference was statistically insignificant. Three patients of the liquor leakage group had diabetes. The standard two-sample test for equality of proportions yields a *p*-value of 0.71. Six patients with ADT were in the single-level group, and 16 patients underwent surgery at two or more segments. The difference was significant (*p* = 0.0139). The ADT rate per segment was 6.18% for single-level operations (97 segments) and 7.4% for multi-level operations (216 segments). The two-sample test for equality of proportion yielded a *p*-value of 0.69654, and therefore no significant difference. No patient suffered from post-operative wound infection, and no patient showed new neurological deficits post-operatively.

**Table 1:** Baseline patient characteristics.

	***n***	**%**
Total number of patients	187	100
1-segment surgery	97	51.87
2-segment surgery	61	32.62
3-segment surgery	22	11.76
4-segment surgery	7	3.74
Previous surgery	12	6.15
Normal weight	30	16.04
Overweight	79	42.25
Obese	78	41.71
Smokers	49	26.2
Diabetics	34	18.19
	**Male**, ***n*** **(%)**	**Female**, ***n*** **(%)**
Gender	72 (38.5)	115 (61.5)
	**Mean**	**Range**
Age (years)	64.27	33–85

**Table 2:** Rate of accidental dural tears (ADTs) in total and by patient subgroups age, BMI groups, smoking status, diabetes, and previous surgery.

	**ADT *n* (%)**	**No ADT (*n*, %)**	**Total (*n*)**	***P***
Total No. of patients	22 (100)	165 (100%)	187	
Age: 33–55 years	4 (2.14)	41 (21.93)	45 (24.06)	0.34
Age: 56–65 years	6 (3.21)	39 (20.86)	45 (24.06)	
Age: 66–75 years	8 (4.28)	37 (19.79)	45 (24.06)	
Age: 76–85 years	4 (2.14)	48 (25.67)	52 (27.82)	
Normal weight	3 (1.6)	27 (14.44)	30 (16.04)	0.92
Overweight	9 (4.81)	70 (37.43)	79 (42.25)	
Obese	10 (5.35)	68 (36.37)	78 (41.71)	
Smokers	4 (2.14)	45 (24.06)	49 (26.2)	0.46
Non-smokers	18 (9.63)	120 (64.17)	138 (73.8)	
Diabetes	3 (1.6)	31 (16.58)	34 (18.18)	0.71
No diabetes	19 (10.17)	134 (71.66)	153 (81.82)	
Previous surgery	7 (58.33)	5 (41.67)	12 (6.42)	<0.001
No previous surgery	15 (8.58)	160 (91.42)	175 (93.59)	

While minor wound healing disorders occurred in 16 (8.6%) of 187 patients [WHD: 1, Hematoma: 9, wound traction blister: 4, dehiscence: 2], we saw no transcutaneous CSF fistulas and no wound infections. None of the patients with ADT developed clinical signs related to low cerebrospinal fluid pressure. No surgeries for wound revision were necessary. Other surgery-associated complications than ADTs were post-operative anemia (6 cases), pneumonia (2), pulmonary embolism (1), urinary tract infection (7), urinary retention (1), and one case of transient ischemic attack (1). Testing for equality of proportions yields that occurrence of perioperative complications was not different within the different age groups (*p* = 0.64) nor within the different BMI groups (*p* = 0.98). Also, smoking (*p* = 0.7) and diabetes (*p* = 0.59) did not increase the complication rate significantly.

## Discussion

ADTs are common intraoperative complications in spine surgery. Reported rates in the literature range from 3.5 to 16.7% in spine surgery for degenerative diseases (10, 14–17). In our study, we saw 22 patients (11.764%) who had experienced an ADT. The likelihood of occurrence of dural tears depends on the type and complexity of the spinal procedure. Tafazal and Sell collected prospective data from 1,549 patients in the United Kingdom and found an ADT rate of 3.5% for discectomy, 8.5% for spinal stenosis surgery, and 13.2% for repeated discectomy (16). Khan et al. reviewed 3,183 patients who underwent surgery for degenerative spine disease and found an ADT rate of 7.6% during patients' first surgery and 15.9% during revision surgery (17). This is in accordance with our findings, as our ADT rate was also significantly higher in patients who underwent previous surgery. Wang et al. reported a rate of 54.4% in patients who had scar tissue from prior surgery (11).

While the influence of previous surgery on the rate of ADTs is well-studied, there is less literature about other predisposing factors for dural tears. In our analysis, with age, BMI groups, smoking status, and diabetes, we focused on clinically relevant parameters readily available in daily practice. It is known that smoking, as well as diabetes mellitus, negatively affects the outcome after spine surgery and can lead to wound healing disorders, surgical site infection, failed fusion, and more re-operations (18–21). However, literature investigating the influence of smoking and diabetes mellitus on the rate of dural tears is rare. Smoking, as well as chronic hyperglycemia, can have various effects on tissue degeneration (22, 23). Diabetes, for example, is known as a non-genetic factor in the pathophysiology of Ossification of the Posterior Longitudinal Ligament (OPLL) (22). However, their role in the degeneration of the ligamentum flavum and other spinal structures is not well-studied. Considering a possible change of tissue elasticity, we wanted to show if either smoking or diabetes increases the risk of dural tears in spine surgery; however, we could not find a significant effect.

A recent study by Smorgick et al. did not find any significant influence of sex, blood loss, BMI, and type of anesthesia, but a significantly higher rate of dural tears in older patients who underwent lumbar laminectomy (24). In a prospective study with 76 patients, Sin et al. found a higher rate of dural leaks in older patients (25). In our ADT group, 13 patients were ≥65 years old (59.09%), and nine (40.90%) were ≤ 64. The difference between the age groups was not found to be significant in our study. When treating older patients, spine surgeons are more likely to be confronted with degenerative spinal problems than when working with younger patients. In the degenerative spine, the probability of experiencing an ADT during surgery appears to be higher. The aging spine dura adheres more strongly to the surrounding structures, especially to the yellow ligament. It has been reported that the yellow ligament texture is related to the rate of ADT occurrence in patients who are undergoing surgery for spinal stenosis. The aging yellow ligament degenerates and loses its elasticity with time. Furthermore, calcium crystals, which are a sign of ossification, are deposited within the ligament (26). Epstein found a marked association between these ossified yellow ligaments and ADT occurrence (7). In Epstein's series of multi-level laminectomies without fusion, ADTs occurred in 31.2% of patients with marked ossified yellow ligaments and in 9.4% of those in whom an ossified yellow ligament extended to the dura. Furthermore, she observed that five of the 10 patients with ADTs suffered from synovial cysts alongside a marked ossified yellow ligament. We saw no synovial cysts in our ADT group.

Published data concerning MAST procedures and ADTs are scarce. Than reported ADTs in seven (6.3%) of 112 patients who underwent MIS procedures (9). Selznick investigated minimally invasive interbody fusion in first surgery and revision surgery and observed a significantly higher overall complication rate in the group that underwent revision surgery (*p* = 0.02). This was primarily due to the higher rate of ADT occurrence [*p* = 0.03; (8)]. Patel compared single-level MIS TLIF with single-level open posterolateral fusion (PLF), as well as PLF plus PLIF (27). He found six ADTs in the conventional surgery cohort (three in the PLF group of 41 patients [7.317%] and three in the PLF plus PLIF group of 42 patients [7.142%]). He saw no ADTs in the MIS group, which consisted of 71 patients. Telfeian et al. (10) reported a durotomy rate of 16.7% in morbidly obese patients who underwent spinal surgery. Cole and Jackson (28) performed minimally invasive lumbar discectomies in 32 obese patients. Incidental durotomies were the most common complication and occurred at a rate of 9.4%. They concluded that the higher rate of ADT was related to the larger working area in obese patients. In contrast, we found no significant differences in the frequency of dural tears within our three BMI groups.

Symptoms caused by ADT, such as nausea, photophobia, vomiting, or transcutaneous cerebrospinal fluid fistulas, can be very limiting for the patient. Literature has suggested a wide range of treatment options for ADT. The recommended period of bed rest ranges from zero to 7 days (9, 29). According to Tafazal and Sell's evaluation (16), 18 of 26 spine surgeons in the United Kingdom advocate post-operative bed rest. Early mobilization has been recommended in a small number of studies. Hodges et al. (30) reviewed 20 patients who had had no bed rest, of whom 25% suffered from symptoms because of CSF leakage. Than et al. (9) investigated the management of incidental durotomies in MISS mobilized patients either immediately after or within 48 h of surgery. None of the patients in Than's study became symptomatic. One reason might be the very small contact with soft tissue in MISS techniques. Because muscles are not resected but dilated to produce a corridor to the spine, muscles will fall back onto the spine after removal of the instruments, and dead space is thereby avoided. The area impacted by this technique may be too small in size to allow for the occurrence of pseudomeningocele, cerebrospinal fluid, and fistula and therefore provides a certain amount of protection. This assumption is congruent with the findings reported by Wong et al. (31). They compared 863 patients who underwent one- and two-level discectomies, foraminotomies, or laminectomies by either MISS or open technique. He saw fewer leakages in the MISS cohort. While no revision was needed in the MISS group, it was in the open group. In our study, we employed a conservative approach to dealing with ADT. In patients in whom ADTs can be closed by sutures and where lesions of the dura are small, we recommended bed rest for two and a half or 3 days. None of the patients to whom this recommendation was made have developed a transcutaneous CSF leak or clinical signs related to low cerebrospinal fluid pressure.

This study was limited by several factors. Due to the observational character and the fact that we only investigated patients who underwent MISS of the study, we cannot compare the ADT rate between conventional surgery and MIS approaches. Due to the patient collective representing daily routine practice, there is a certain heterogeneity of the study population, e.g., when considering the number of fused levels. Another limiting factor is the design as a single-center study. Compared to multicenter studies, results and effects might be over- or underestimated. Therefore, future studies should be conducted as multicenter studies and focus on the exact reasons why MISS might lead to a low rate of CSF fistulas and associated clinical signs.

## Conclusions

According to our results and those published in the literature, the essential advantages of MISS techniques range from minimized soft tissue damage, reduced blood loss, diminished post-operative pain to a shorter length of stay in hospital (1, 2, 4, 32, 33). The frequency of ADT occurrence in this present investigation is similar to that reported for open spinal surgery. Previous spinal surgery increased the risk for ADTs significantly. To our knowledge, ours is one of the largest single-center studies. We also believe that the rather small corridor to the spine created by MISS instruments provides a certain amount of protection against pseudomeningocele or cerebrospinal fluid fistulas. As we did not see any post-operative CSF fistulas or symptoms associated with low CSF pressure, we recommend early mobilization in case of ADTs in MISS.

## Data Availability Statement

The raw data supporting the conclusions of this article will be made available by the authors, without undue reservation.

## Ethics Statement

The studies involving human participants were reviewed and approved by Ethics Committee of the Federal State of Lower Austria. The patients/participants provided their written informed consent to participate in this study.

## Author Contributions

SA and WS designed the study and wrote the manuscript. CR, CL, NS, and WT collected, assembled, and analyzed the data. Project planning was performed by AG, HS, MA, KA-H, and WS. All authors read, edited, and approved the manuscript.

## Conflict of Interest

The authors declare that the research was conducted in the absence of any commercial or financial relationships that could be construed as a potential conflict of interest.
